# Programmed Catalytic Therapy-Mediated ROS Generation and T-Cell Infiltration in Lung Metastasis by a Dual Metal-Organic Framework (MOF) Nanoagent

**DOI:** 10.3390/pharmaceutics14030527

**Published:** 2022-02-27

**Authors:** Bhanu Nirosha Yalamandala, Pin-Hua Chen, Thrinayan Moorthy, Thi My Hue Huynh, Wen-Hsuan Chiang, Shang-Hsiu Hu

**Affiliations:** 1 Department of Biomedical Engineering and Environmental Sciences, National Tsing Hua University, Hsinchu 30013, Taiwan; bhanunirosha@gmail.com (B.N.Y.); chen0922102113@gmail.com (P.-H.C.); m.3nayan@gmail.com (T.M.); myhue100294@gmail.com (T.M.H.H.); 2 Department of Chemical Engineering, National Chung Hsing University, Taichung 402, Taiwan; whchiang@dragon.nchu.edu.tw

**Keywords:** drug delivery, nano-catalytic medicine, MOF, autophagy, immune response, lung metastasis

## Abstract

Nano-catalytic agents actuating Fenton-like reaction in cancer cells cause intratumoral generation of reactive oxygen species (ROS), allowing the potential for immune therapy of tumor metastasis via the recognition of tumor-associated antigens. However, the self-defense mechanism of cancer cells, known as autophagy, and unsustained ROS generation often restricts efficiency, lowering the immune attack, especially in invading metastatic clusters. Here, a functional core-shell metal-organic framework nanocube (dual MOF) doubling as a catalytic agent and T cell infiltration inducer that programs ROS and inhibits autophagy is reported. The dual MOF integrated a Prussian blue (PB)-coated iron (Fe^2+^)-containing metal-organic framework (MOF, MIL88) as a programmed peroxide mimic in the cancer cells, facilitating the sustained ROS generation. With the assistance of Chloroquine (CQ), the inhibition of autophagy through lysosomal deacidification breaks off the self-defense mechanism and further improves the cytotoxicity. The purpose of this material design was to inhibit autophagy and ROS efficacy of the tumor, and eventually improve T cell recruitment for immune therapy of lung metastasis. The margination and internalization-mediated cancer cell uptake improve the accumulation of dual MOF of metastatic tumors in vivo. The effective catalytic dual MOF integrated dysfunctional autophagy at the metastasis elicits the ~3-fold recruitment of T lymphocytes. Such synergy of T cell recruitment and ROS generation transported by dual MOF during the metastases successfully suppresses more than 90% of tumor foci in the lung.

## 1. Introduction

Metastasis is responsible for over 90% of cancer-related deaths [1,2]. It is developed in the primary tumor in which the cancer cell escapes from the immune system and forms the secondary tumor at distant organs [3,4,5]. Recently, immune therapy held promise to suppress the metastatic cells via the cytotoxic T lymphocytes [6,7,8]. Despite the recent advance in immunotherapy, the invading clusters of metastases usually smaller than 100 mm^3^ poorly performed vascularization and restricted physical contact of T lymphocytes, lowering immune responses [9,10,11,12]. Furthermore, the invasion of secondary tumors fills up the lymphatic vessels and reduces the space to recruit immune cells [13,14]. Thus, the recruitment of cancer-specific T lymphocytes during metastasis is critical.

To elicit T cells, a potential solution is to eliminate the cancer cells to generate a high number of cancer-associated antigens. In this regard, the reactive oxygen species (ROS), which plays a typical role in the cellular signaling-derivatized oxygen molecules, served as an oxidative damager in excessive expression (specifically H_2_O_2_), and could lead to potent oxidative harm within cancer cells [15,16]. Such ROS-responsive biological targets have attracted great attention in the redox chemistry of cancer therapeutics via promoting the intracellular H_2_O_2_ conversion to •OH [17,18,19]. It can induce a Fenton-like reaction in the tumor in which H_2_O_2_ is disproportionated to toxic •OH, facilitating oxidization and damaging intracellular proteins and organelles [20]. However, the failure of proteins and organelles could simulate the genotoxic reaction and metabolic insufficiency [21], leading to the activation of autophagy (also known as the self-defense mechanism) [22,23]. In this mechanism, the cells engulf cytoplasmic organelles in autophagosomes and transport them to lysosomes for degradation. This helps the clearance of •OH-damaged proteins and organelles for detoxification [24,25].

To mitigate the activation of autophagy at tumors, different modified-MOFs have been developed as light- or sono-enhanced Fenton reactions [26,27]. The light-responsive materials possessing iron (Fe^2+^ and Fe^3+^) under a NIR light or radiation were able to trigger the hydrogen peroxide to effectively produce hydroxyl radicals when compared to the traditional Fenton methods [28,29]. Such ROS generation by photo-reduction of Fe^3+^ ensures the proceeding of the reaction. For example, Hu et al. reported that the Fe^2+^-loaded lanthanide-doped porous particles under NIR irradiation to cause Fe^2+^ to produce localized •OH radicals in cancer mitochondrion, showing the strong mtDNA damage [27]. In blocking the autophagic flux, chloroquine (CQ) has been developed as a classical inhibitor of autophagy [30]. The innate immunity provides downstream regulation of autophagy by activating the receptor that further enhances the production of cytokines and phagocytosis [31,32,33]. Conversely, in adaptive immunity, an increased source of antigens through autophagic activation promotes the CD8^+^ T cells for direct cytotoxicity in cancer metastasis [33]. The autophagy activation enhances the recruitment of LC3B protein (microtubule-associated protein 1 light chain 3B), the fusion between phagosomes and lysosomes leading to the increased antigen presentation for adaptive immunity [34]. Thus, the autophagic inhibition is required for preventing cargo degradation, which may help to promote the T cell infiltration in metastatic tumor suppression.

**Scheme 1:**
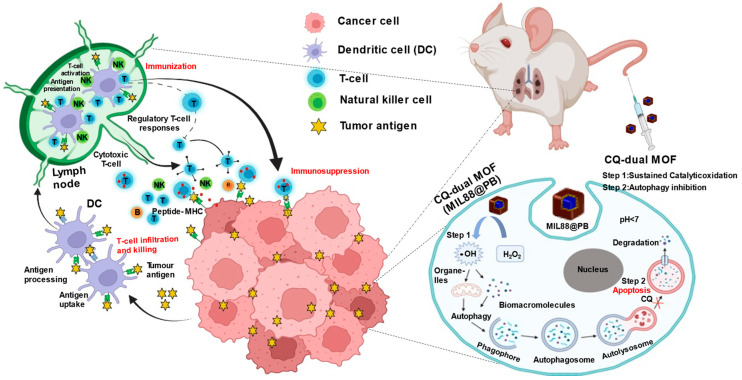
Schematic illustration of dual MOF catalytic activity and immune response. The dual MOF programs peroxide mimic in the cancer cells and sustain to generate ROS via the core-shell characteristics (step 1). With a high cellular uptake, chloroquine (CQ) served as an inhibitor of autophagy, and regulates the autophagy flux by de-acidifying the lysosomes, lowering the self-defense mechanism of cancer cells (step 2). The accumulation of dual MOF in lung metastasis promotes the accumulation of specific T-cell responses (CD4^+^ and CD8^+^).

Here, a core-shell metal-organic framework nanocube (dual MOF) that integrated the features of programmed ROS and autophagy inhibition was developed for eliciting T cells towards tumor metastasis (Scheme 1). The dual MOF is able to program peroxide mimic in the cancer cells and can be sustained to generate ROS via the core-shell characteristics (step 1). With a high cellular uptake, the chloroquine (CQ) serves as an inhibitor of autophagy, and regulates the autophagy flux by de-acidifying the lysosomes, lowering the self-defense mechanism of cancer cells (known as autophagy) and boosting the intracellular oxidative damage (step 2). To trigger the therapeutic processes, NH_2_-MIL-88B (Fe) as shell has been chosen as a highly efficient catalysis to guarantee the upstream generation of the amount of •OH radicals, whereas Prussian blue (PB) as a core shows catalytic activity for the reduction of H_2_O_2_. The effective catalytic and dysfunctional autophagy at the metastasis could elicit the infiltration of T lymphocytes. The versatile dual MOF is an excellent ROS generator to actuate cancer cell death and enhance T cell recruitments for immune therapy.

## 2. Method

### 2.1. Materials

Polyvinylpyrrolidone (PVP, average molecular weight (MW) = 50,000 g/mol, Sigma-Aldrich, ST.Louis, USA), hydrochloric acid (HCl, 36.0%, Sigma-Aldrich, ST.Louis, USA), potassium ferricyanide (K_3_[Fe(CN)_6_], Sigma-Aldrich, ST.Louis, USA), pluronic (F127, Sigma-Aldrich, ST.Louis, USA), iron (III) chloride hexahydrate (FeCl_3_·6H_2_O, Sigma-Aldrich ST.Louis, USA), acetic acid (CH_3_COOH), and aminoterephthalic acid (NH_2_-BDC, Thermo Fisher Scientific, Lancashire, UK). Chloroquine (CQ) was obtained from Sigma-Aldrich. Aqueous solutions were prepared with deionized (D.I.) water (17.7 MU cm) produced from Milli-Q water purification. All other chemicals used in this work were obtained from commercial suppliers which were of analytical grade and used without any further purification.

### 2.2. Synthesis of PB MOF

The synthesis procedure of Prussian blue (PB) was carried out via a modified approach developed by Yamauchi group [35]. In a typical process, 3 g of PVP and 0.2267 g of K[Fe(CN)_6_] were dissolved into 40 mL D.I. water under vigorous stirring and then 35.0 µL of HCl was added to form a clear solution. The resulting solution was continuously stirred for 30 min and placed into an autoclave at 80 °C for 20 h. The mixture was washed many times with ethanol and the resultant product was dried by particle lyophilization.

### 2.3. Synthesis of NH_2_-MIL88

The synthesis procedure of NH_2_-MIL88B (Fe) was carried out via a hydrothermal route by dissolving 0.1783 g of FeCl_3_ and 0.16 g of F127 in 15 mL of D.I. water. After 45 min of stirring, 150 µL of acetic acid was added to regulate the size of nanoparticles followed by another 45 min of stirring. Then, 60 mg of NH_2_-BDC was added to the mixture for the further stirring of 2 h, and the resulting mixture was transferred to an autoclave at 110 °C for 16 h. After aging, the mixture was washed several times with D.I. water and the resultant was obtained by particle lyophilization.

### 2.4. Synthesis of MIL88@PB (Dual MOFs)

The dual MOFs were synthesized via the hydrothermal route by coating the PB with NH_2_-MIL88B (Fe) MOF as an outer shell through a layer-growth method. Briefly, 17.83 mg of FeCl_3_·6H_2_O and 3.75 mg of F127 were added to 4 mL of D.I. water, and this mixture was further added to 28 mL of PB solution (0.00813 g/mL). After 1 h of stirring, 37.5 µL of acetic acid was added, followed by another 1 h of stirring. Then, 6 mg of NH_2_-BDC was added. After continuous stirring of 2 h, the resulting mixture was autoclaved at 110 °C for 16 h. After aging, the mixture was washed by ethanol and D.I. water for three times, and the precipitates were collected. The final product was obtained by particle lyophilization.

### 2.5. Characterizations

The morphologies of PB, MIL88 and MIL88@PB nanoparticles were analyzed by field emission scanning electron microscopy (FE-SEM) and cryo-high-resolution transmission electron microscope (Cryo-HRTEM, JEM-2010, JEOL, Tokyo, Japan). Nanoparticle’s diameter and surface charge were analyzed by dynamic light scattering (DLS, Nano-ZS, Malnern, Malvern, UK). Powder X-ray diffraction (XRD) patterns were tested on a Rigaku Japan TTRAX III equipped with Cu Ka radiation of 2 theta range. Fourier-Transform infrared (FTIR) spectrum was analyzed in the range of 500–4000 cm^−^^1^ with a resolution of 4 cm^−^^1^.

### 2.6. In Vitro Drug Release

To load CQ into the dual MOF nanoparticles, the nanoprecipitation method with 2 mg of PB, MIL88, and dual MOFs dissolved into 15 mL of PBS solution by adding 1.8 mg of CQ. The drug release test was carried out by collecting 2 mL of supernatant by washing the mixture for UV-vis absorption (The Evolution 350 UV-Vis Spectrophotometer, Therma Fisher Scientific). The mixture was replaced with fresh 2 mL of PBS solution. The experiment was carried out for 50 h to measure the drug encapsulation efficiency.

### 2.7. In Vitro Cellular Toxicity

A total of 100 µL of B16F10 (a murine tumor cell line; skin melanoma cells) cells at a density of 1 × 10^4^ cells per well were seeded into 96-well plates using standard cell medium (DMEM) and incubated for 24 h in 5% CO_2_ at 37 °C. Then, the cells were treated with 100 µL of MOF nanoparticles at different concentrations (12.5, 25, 50, 75 and 100 µg/mL) for another 24 h. Cells were washed carefully three times with PBS solution and cell cytotoxicity assay was carried out by adding 10 µL of MTT Presto blue solution to each well incubated for 10–15 min before. Finally, a plate reader (Synergy HT Multidetection microplate reader, BioTek Instruments, Inc., Santa Clara, CA, USA at a wavelength of 570 nm) was used to measure the absorbance of each well and expressed the percentage of cell viability.

### 2.8. In Vitro Cellular Uptake

For the cellular uptake experiment, 20 µL of quantum dots (QDs) was loaded into PB, MIL88, and dual MOF with different concentrations (75, 100, 200 and 400 µg/mL). The QD-labeled MOFs were added to 1 × 10^5^ of B16F10 cells which were seeded on coverslip in 6-well plates for 24 h. The medium was replaced with 1 mL of MOFs solution and further incubated for 24 h at 37 °C. Then, the staining method is carried out by replacing the 1 mL of medium with 3% formaldehyde solution to fix the cells for 30 min of incubation. Subsequently, the cell solution was removed, and 1 mL of Triton (0.1%) was added for 30 min of incubation for permeabilization. Finally, the cell nuclei and cytoskeleton were stained by 1 mL of DAPI (1 μg/mL) for 20 min and F-actin (300 units/mL) overnight at 37 °C. Between each step, the cells were washed carefully with PBS three times. The cells were mounted on glass slides and observed by confocal laser scanning microscopy (CLSM Zeiss LSM 800, Oberkochen, Germany).

### 2.9. In Vitro LC3B Autophagy Protein Expression (Regulation of Autophagosomes)

LC3B, an autophagy protein on autophagosome, expression was evaluated. CQ-loaded PB, MIL88, and dual MOFs at different concentrations were treated to B16 cells to investigate the activity of autophagosomes and fusion with lysosomes. The QD-labeled MOFs were added to 1 × 10^5^ green fluorescence-expressed B16F10 (GFP-B16F10) cells, which were seeded on a coverslip in 6-well plates for 24 h. The medium was replaced with 1 mL of MOF solution and further incubated for 24 h at 37 °C. Then, the staining method was carried out by removing the 1 mL of medium and adding 3% formaldehyde solution to fix the cells for 30 min incubation. After that, the cell solution was replaced with 1 mL of Triton (0.1%) and 20–30 min incubation for permeabilization. The cell nuclei and cytoskeleton were stained with 500 µL per well with DAPI (1 μg/mL) and F-actin (300 units/mL) for 2 h, and then, 1 mL of LC3B primary antibody was added with 5% of blocking buffer bovine serum albumin (BSA) overnight at 4 °C, respectively. Finally, secondary antibody (LC3B) was added with blocking buffer (BSA) for 1 h of incubation. The cells were mounted on glass slides and tested the MOF treated autophagosome activation by Confocal laser scanning microscopy (CLSM Zeiss LSM 800, Oberkochen, Germany).

### 2.10. In Vitro Catalytic Performance of Dual MOFs

The catalytic performance of MOFs by detection of •OH radicals in vitro was evaluated by demising methylene blue (MB) method. It was tested at pH 6.4 and 7.4 by adding 100 µg/mL of PB, MIL88, and dual MOFs into 250 µL of glutathione (GSH, 40 µM) and 100 µL of H_2_O_2_ solution. Methylene blue of 50 µL (500 µM) was added to detect the ROS generation in addition to MOF. The MOF catalytic activation was measured by UV-Vis Absorption. The experiment was conducted for 4 h to detect the initiation of Fenton reaction and catalytic activation of MOFs.

### 2.11. Tissue Section Immunostaining

Animal study and surgical procedures were performed in accordance with the protocol approved by the Institutional Animal Care and Use Committee (IACUC), National Tsing Hua University, Hsinchu, Taiwan (IACUC protocol and approval number are 10704). Female C57BL/6 mice of 6 to 8 weeks old (purchased from National Laboratory Animal Center, NLAC, Taiwan) were adopted as the animal model of tumor growth inhibition experiment. For the development of mouse lung metastasis, 1 × 10^6^ GFP-B16F10 cells were trypsinzed, washed, and suspended in DMEM in advance. Then, 100 µL of 1 × 10^6^ GFP-B16F10 cells resuspended with PBS was injected to the mouse intravenously. After 13 days of cancer cell injection, the QD-labeled PB, MIL88, or dual MOF nanoparticles were injected into the mice. Then, 24 h post injection, the mouse was sacrificed, and lung tissue was surgically excised to make a frozen section. The organs were perfused in PBS and further transferred to 4% paraformaldehyde overnight at 4 °C. Subsequently, they were fixed in OCT gel at −80 °C for frozen section slicing. Dehydrated frozen tissue was carried by immersing it for 10 min with 100% methanol. The slices were washed with PBS (Gibco, 10010023) to remove the OCT residue. Before staining, 100 μL of blocking buffer (BSA) was added and incubated for 1 h at room temperature. Then, 100 µL of diluted primary antibody CD8 (Rabbit-anti CD8 α, Abcam, ab217344, 1:1000 dilution) and CD4 (Rat-anti CD4, Abcam, ab25475, 1:1000 dilution) were added and incubated overnight at 4 °C. After 24 h, the secondary antibody CD8 (Donkey anti-rabbit 488, Jackson, 112545143) and CD4 (Donkey anti-rat 647, Jackson, 112605167) were added to samples for another 1 h of incubation. Between each step, the slices were washed for three times carefully with 1× PBS solution through an autoshaker. DAPI mounting medium (Abcam, ab104139) was used and the slide was sealed with nail polish. The tissue images were captured with Confocal laser scanning microscopy (CLSM Zeiss LSM 800, Oberkochen, Germany).

### 2.12. In Vivo Flow Cytometry Analysis

The in vivo flow cytometry analysis was plied to examine T cells in lung metastasis. Briefly, 100 µL of 1 × 10^6^ GFP-B16F10 cells resuspended with PBS was injected to the mouse intravenously. For the treatment groups, the NPs were injected via a 27-gauge needle through a tail vein 13 days post implantation of a tumor. After 24 h, the mice were sacrificed on the 14th day and the lungs were dissected for immune staining. The isolated tissues were mechanically disrupted and were added into the RBC lysis buffer for lyses red cells. Then, to attain tumor single-cell suspensions, enzymatic digestion in 0.1 mg/mL collagenase solution (Sigma, C0130), 0.1 mg/mL Liberase solution (TL Roche), 1 μg/mL DNase solution (Sigma, DN25), and 6.6 μg/mL dispase I solution (Sigma, D4818) in HBSS buffer (Sigma, H8264) for 30 min. The suspension was filtered through a 70 μM filter after digestion to remove the cellular debris. For B16F10 cells, the formed single-cell suspension was then stained with fluorochrome-labeled antibodies were analyzed by flow cytometry after the surface staining for 30 min at 4 °C. The characterization of the T cell subsets was performed using fluorochrome conjugated anti-mouse Abs: anti-CD4 PE (BD, 553730), anti-CD3e FITC (BD, 553062), anti-CD8a APC (BD, 553035), and anti-CD45 PE-Cy7 (BD, 552848). The isolated spleen was mechanically disrupted and added into RBC lysis 40 buffer for lyses red cells. Then, to attain tumor single-cell suspensions and to remove debris, the suspension was filtered through a 70 μM cell strainer. The characterization of the matured DC cell subsets was performed using antibody anti-mouse CD11c-FITC (BD, 553801), anti-Mouse CD80-APC (BD, 560016), and anti-MouseCD86-PE-Cy7(BD,560582). Data were acquired using a BD FACSAriaTM II flow cytometer (Invitrogen, Thermo Fisher Scientific, Oregon, USA) and analyzed with Flow Jo software (version 7.6.1).

## 3. Results and Discussion

### 3.1. Characterization of PB, MIL88, and Dual MOF Nanoparticles

**Figure 1:**
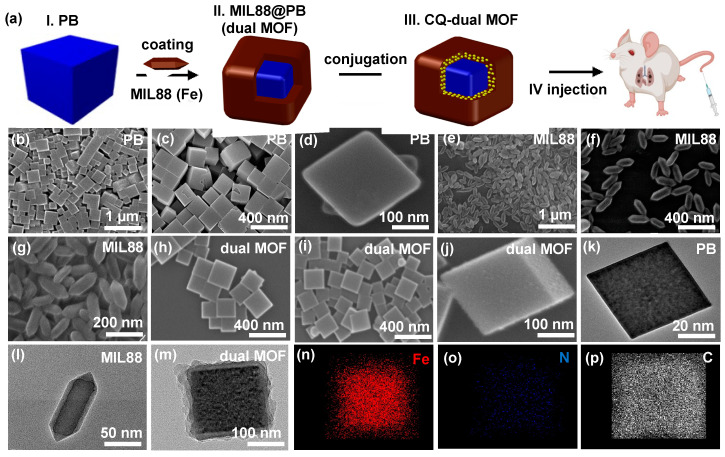
Synthesis and characterizations of dual MOFs. (**a**) The schematic illustration of the synthesis process of CQ-loaded dual MOF (CQ-dual MOF). The dual MOF system was reached by coating the inner Prussian blue (PB) with an outer MIL88 as a metal source and NH_2_BDC as an organic linker while. F127 pluronics and acetic acid were used for stability and tailoring the size of the MOF. (**b**–**i**) SEM and (**j**–**m**) TEM images of PB, MIL88, and dual MOFs. (**n**–**p**) Element mapping analysis of dual MOFs.

The dual MOFs mainly consisted of two elements, PB nanocubes and MIL88 shells, as shown in Figure 1a. To investigate the morphologies of resulting particles, scanning electron microscopy (SEM) and transmission electron microscopy (TEM) were applied. SEM and TEM images reveal the surface characteristics of PB, MIL88, and MIL88@PB with their morphologies of cubes and bipyramidal hexagons (Figure 1b–m). The sizes of PB nanocubes were ranged from 200 to 260 nm, showing a smooth and sharp surface on its structure (Figure 1b–d). As revealed in Figure 1e–g, MIL88 exhibited monodisperse bipyramidal hexagons with an average length of 130 nm. After MIL88 coating, dual MOFs could maintain the cubic structures as PB (Figure 1h,i), but a clear shell constructed by MIL88 was observed in TEM analysis (Figure 1m). The well-coating of MIL88 on PB was potentially attributed to the affinity of molecule absorption between PB and MIL88 precursors. The evidence from elemental mappings also indicated that the elements of Fe, N, and C were distributed on the dual MOFs (Figure 1n–p), implying the successful coating of two materials.

**Figure 2:**
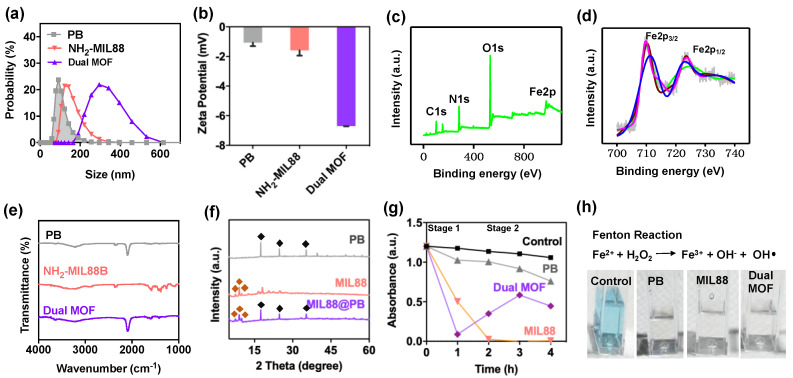
Characterizations of single and dual MOFs. (**a**) Size distribution and (**b**) zeta potential ofPB, MIL88, and dual MOFs. (**c**) XPS spectrum of all the elements and (**d**) Fe2p spectrum of dual MOFs, demonstrating all the iron components in the trivalent state. (**e**) FTIR and (**f**) X-ray diffraction spectrum of PB(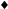), MIL88(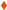), and dual MOF. Slight disappearances reflections of 2-Theta and intensities in the dual MOF were due to the shielding effect of MIL88 on PB. (**g**,**h**) Catalytic performance of control, PB, MIL88, and dual MOF.

The growth of dual MOF also reflected the size distribution of PB, MIL88, and dual MOFs, where the hydrodynamic diameter of PB, MIL88, and dual MOFs displayed 144, 185, and 296 nm in agreement with the DLS technique, respectively (Figure 2a). The zeta potential results of PB, MIL88, and dual MOFs showed the surface charge of −1.07, −1.59, and −6.53 (Figure 2b). The lower surface charge of dual MOF was probably partly caused by the defects of MIL88, since the crystallinity of MIL88 on PB was decreased (as demonstrated on TEM, Figure 1m). Furthermore, the chemical bonds of dual MOFs, representing the binding energy at 711 and 721 eV corresponding to the Fe2p_1/2_ and Fe2p_3/2_, suggested that all Fe atoms in dual MOFs were in the trivalent state specifying their interactions with the organic ligand, 2-Aminoterephtalic acid (Figure 2c,d). Furthermore, Fourier-transform infrared (FTIR) spectrum results revealed that a strong peak at 2090 cm^−^^1^ (Figure 2e) in PB and dual MOF, indicating the stretching absorption of CN bands from the PB. The absorption bands at 1156–1096 cm^−^^1^, which is attributed to the stretching vibration of C-O in the backbone of Pluronic F127 (Figure 2e, MIL88). Four absorption bands at 1585, 1495, 1436, and 1381 cm^−^^1^ are attributed to the symmetric and asymmetric vibrations of carboxyl groups. In addition, two bands at 3466 and 3363 cm^−1^ could be observed as the characteristics of symmetric and asymmetric stretching absorptions of primary amine groups of NH_2_-BDC linkers. Meanwhile, dual MOF displayed the functional groups of inner PB layer and outer MIL88, respectively. Next, the crystal structures of three particles were evaluated by X-ray diffraction (XRD, Figure 2f). XRD analysis of PB exhibited the major diffraction planes at (200), (220), and (400), which are the characteristic of the PB crystal planes indexed face-centered cubic lattice (Figure 2f in symbol diamond). After coating with MIL88, dual MOF revealed both the diffraction pattern of PB and MIL88 without any changes, indicating the slight effects of coating process in crystallinity. The lower XRD intensity of PB in dual MOF was caused by the shield effects of MIL88 on PB. The MOF is an instable material in the presence of water. In our study, the preservation of materials was carried out by the drying process under a vacuum to avoid the hydrolysis. The hydrolysis could lead the breaking of Fe–O bonds and exposing more =C–H groups [36]. The colloidal stability of PB, MIL88, and dual MOF in cell culture medium (DMEM supplemented with 10% FBS serum) for 24 h were also estimated to understand the process (Figure S1). The variation of size and surface charge could be observed in both MIL88 and dual MOF, indicating the hydrolysis effect.

The catalytic performance of PB, MIL88, and dual MOFs were investigated by the detection of •OH radicals. The experiments via adding MOFs into H_2_O_2_ solution were performed for 4 h at pH 6.4 and 7.4, respectively. Methylene blue (MB), a detecting dye, is added to evaluate ROS generation in the reaction mixture to understand Fenton reaction. Generally, the iron-based MOF reacted with H_2_O_2_ turn to ferric ions and release •OH radicals, facilitating the MB reaction and turning it into reaction color. This also confirms that MOFs can catalytically activate H_2_O_2_ to produce •OH radicals. At pH 6.4, MIL88 initiated the Fenton reaction immediately and consumed all ferric ions within 1 h, exhibiting no higher reaction ability over time (Figure 2g,h). Compared to MIL88, PB exhibited the slower Fenton reaction rate and sustained generation of ROS for four hours. Dual MOF performed the programmed ROS generation to manipulate the Fenton reaction, which demonstrated the strong Fenton reaction in the first hour and sustained reaction for another 3 h. The dual MOF catalytic activation happened through the various dynamics of electron transfer initiated by Fenton reaction in MIL88 and PB. We further tested the MOF catalytic activity at pH 7.4, which showed no significant reaction; only a weak Fenton reaction occurred.

### 3.2. In Vitro Drug Release

To measure the encapsulation efficiency, the supernatant of loading drug was collected by centrifugation of the mixture. The CQ loading efficiency was calculated by measuring the initial drug and released drug in supernatant with time different points. Due to the MOF having the porous structures with the affinity to CQ via the p-p interactions and van der Waal forces, the resulting loading efficiency reached about ~70%. The loading content was kept in a similar concentration at 1 mg/g particles for three types of particles for the comparison of treatment effects. The release profiles were shown in Figure S2, which displayed the sustained release patterns for each group.

### 3.3. In Vitro Cellular Uptake and Cytotoxicity of PB, MIL88, and Dual MOF

**Figure 3:**
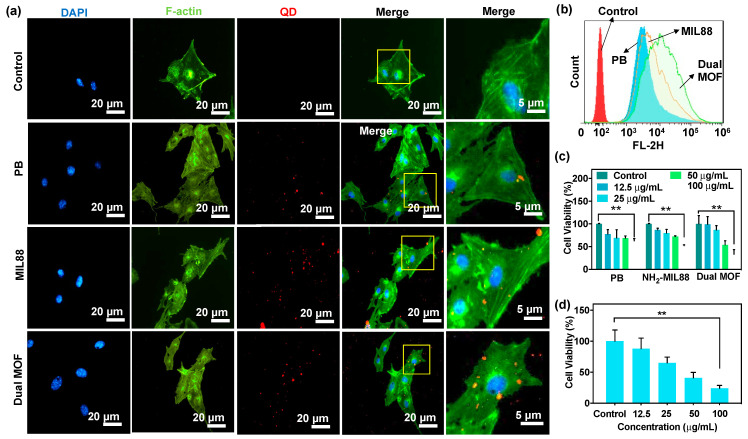
(**a**) CLSM images of cellular uptake of B16F10 incubated with PB, MIL88, and dual MOF labeled by QDs. Blue, red, and green fluorescence represent nucleus staining with DAPI, particle staining with QDs, and cytoskeleton staining with F-actin, respectively. (**b**) Flow cytometry analysis of PB, MIL88, and dual MOF after 24 h of incubation in B16F10 cells. (**c**) Cell viability of B16F10 cells in the presence of PB, MIL88, and dual MOF solution at various concentrations. (**d**) Cytotoxicity of B16F10 cells incubated with CQ-loaded dual MOF at various concentrations. Quantitative significant statistical data were calculated via Student’s *t*-test, ** *p* < 0.01.

PB, MIL88, and dual MOF were incubated with B16F10 (a murine tumor cell line; skin melanoma cells) for 24 h to verify the cell uptake. CdSe quantum dots (QDs) was loaded into the particles by the hydrophobic interaction for tracking their intracellular behaviors. After 24 h of incubation, CLSM was applied to evaluate the cellular uptake, in which the nucleus and cytoskeleton were stained by DAPI and F-actin, respectively. CLSM images of B16F10 cells in Figure 3a displayed that only few PBs were observed in the cytoplasm. For MIL88 and dual MOF groups, the amounts of particles in the cells were higher than PB group. As documented in literature, the rod-like nanoparticles (such as MIL88) with the anisotropic properties could enhance the internalization efficiency in a lying-down or standing-up manner [37]. On the other hand, dual MOF possessed the negative charges which improved the nonspecific interactions with the plasma membrane [38,39]. Therefore, the two types of particles improved the cell uptake efficiency. The results of flow cytometry were also used to quantify the cell uptake in Figure 3b, indicating the stronger accumulation of MIL88 and dual MOF in B16F10.

The cytotoxicity of PB, MIL88, and dual MOF was examined by incubating particles with B16F10 (a murine tumor cell line; skin melanoma cells) at various concentrations for 24 h. The in vitro cell cytotoxicity studies of three groups were tested by using MTT assay. The results demonstrate that the dual MOFs showed higher cytotoxicity compared to the remaining groups. Compared to the control group, all the three groups increase the cytotoxicity when increasing MOFs concentration (Figure 3c). Conversely, the dual MOF group at 100 µg/mL showed significant cytotoxicity compared to the PB and MIL88 groups, which might be caused by programmed ROS generation effects of dual MOF. The outer coating shell, i.e., MIL88, not only had the ability of loading CQ but also increased the cell cytotoxicity (Figure 3d).

### 3.4. In Vitro Cellular Uptake and LC3B Protein Expression (Regulation of Autophagosomes)

**Figure 4:**
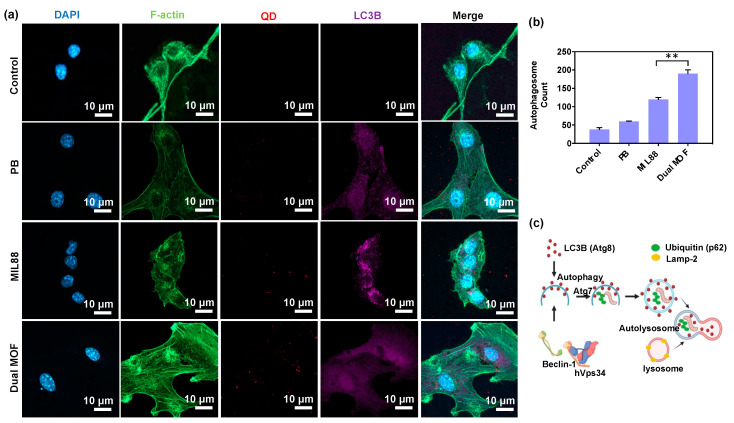
Autophagy activation through LC3B protein expression. (**a**) Confocal fluorescence images of B16F10 cells incubated with PB, MIL88, and MIL88@PB (100 µg/mL) (blue, green, and red fluorescence represent nucleus, cytoskeleton, and NP staining with DAPI, F-actin, and QD, respectively; purple fluorescence represents LC3B expression, which shows the quantity of autophagosomes), the experiment was conducted three times on three independent 6-well plates. (**b**) Autophagosome quantification of PB, MIL88, and MIL88@PB by image J analysis software. (**c**) Schematic representation showing increased LC3B expression leads to increased autophagy expression. Quantitative significant statistical data were calculated via Student’s *t*-test, ** *p* < 0.01.

LC3B, an autophagic marker, also known as Atg8 protein, is associated with complete autophagosome formation. Therefore, increased LC3B is a good indication for autophagososme formation [30], and LC3B protein expression reflects the regulation of autophagosomes. While MOFs entered the tumor region, the cancer cells interacted with the ferric ions of MOFs could trigger the initiation of Fenton reaction, converting excessive intrinsic non-toxic H_2_O_2_ into highly oxidative •OH radicals. The release of •OH radical attacks and inactivates organelles and proteins. On this basis, autophagic responses are activated through the recruitment of LC3B proteins to degrade the proteins and organelles. To confirm the recruitment, LC3B protein expression of B16F10 treated by PB, MIL88, and dual MOFs were evaluated by CLSM. As shown in Figure 4a, CLSM images demonstrated that three groups (PB, MIL88, and dual MOFs) can actuated the LC3B expression when compared to control group, whereas dual MOFs showed slightly higher expression of LC3B compared to other groups, indicating the activation of autophagososmes. Furthermore, the expression of LC3B quantified by Image J software also reflected the results (Figure 4b). As documented in the literature, pre LC3B cleaved to form LC3-I by Atg4 and activated by Atg7 protein to form LC3-II. An increased expression of LC3-II demonstrates increased autophagy [40]. Autophagy-regulated proteins (LC3B/Atg8, Beclin-1/Atg6, Atg7, Lamp-2, p62, and aggregates of polyubiquitin) in autophagosome formation and lysosomal fusion (Figure 4c).

### 3.5. CQ Inhibition of LC3B Protein Expression

**Figure 5:**
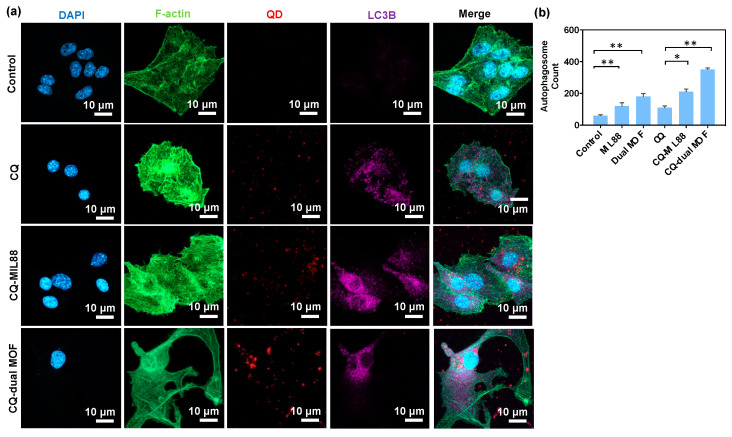
Autophagy regulation by CQ, CQ-MIL88, and CQ-dual MOF. (**a**) Confocal florescence images of B16F10 cells incubated with CQ, CQ-MIL88, and CQ-dual MOF (100 µg/mL). Purple florescence represents LC3B expression, which shows the quantity of autophagosomes. Blue, green, and red fluorescence represent nucleus, cytoskeleton, and NP staining with DAPI, F-actin, and QD, respectively. The experiment was conducted for three times on three independent 6-well plates. (**b**) The quantification of autophagosome of B16F10 cells incubated with CQ, CQ-MIL88, and CQ-dual MOF (100 µg/mL) by image J analysis software. Quantitative significant statistical data were calculated via Student’s *t*-test, * *p* < 0.05, ** *p* < 0.01.

Having demonstrated the LC3B protein expression in B16F10 cells, the co-delivery of autophagy inhibitor (CQ) in MOFs was also investigated. The experiment was performed with dual MOFs, CQ alone, and CQ-dual MOF groups. CLSM images in Figure 5a exhibited the strong fluorescence expression of LC3B (purple fluorescence) in MIL88 and Dual MOFs groups once the particles co-delivering CQ. Compared to particles’ groups, the CQ along group revealed the slightly LC3B expression, which might be caused by the autophagy inhibition effect. After quantifying the results, the CQ-loaded dual MOF groups demonstrated the increased autophagosome numbers (Figure 5b). Based on these results, the amounts of autophagosomes were not only affected by the sustained ROS generation but also the inhibition of degradation of resulting autolysosomes. As the literature documented, the unprotonated CQ can be soluble and diffuse freely in cell membranes and organelles [41,42]. When protonated in an acid environment, such as the lysosomal compartment, CQ would be trapped since the protonated form is insoluble in a lipid membrane. Because of protonation and accumulation of CQ in the lysosomal compartment, it would deplete hydrogen ions and change the internal environment, leading to blocking the degradation substrates in lysosomes and stopping the metabolization. This would inhibit normal lysosomal functioning, which has been demonstrated to be the main reason for blocking the fusion of autophagosomes with lysosomes [41,42]. The acidity-tropic nature of CQ promotes its accumulation and trapping in cancer cells, further facilitating its pharmacological autophagy inhibition. Such blocking effects by CQ combined with MOF on catalytic ROS generation, controlling the degradation of damaged cellular organelles, which leads to the oxidative damage induced by nanocatalytic therapy, and a significant increase in autophagosomes.

### 3.6. In Vivo Study of Mice Bearing Lung Metastasis Treated with Dual MOF

**Figure 6:**
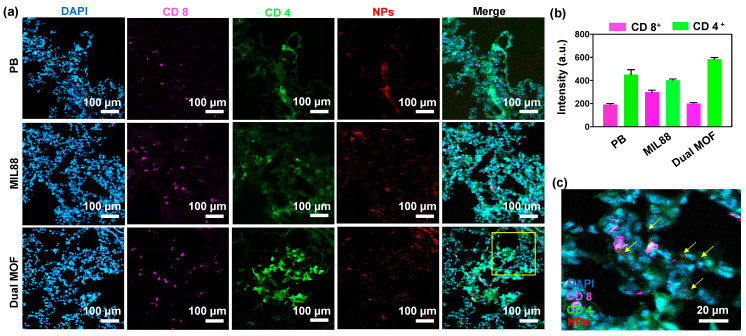
In vivo study of mice bearing B16F10 lung metastases treated with PB, MIL88, and dual MOF. (**a**) CLSM images of lung expressing CD4^+^ and CD8^+^ after treated with PB, MIL88, and dual MOF. Purple and green florescence represents CD4^+^ and CD8^+^ expressed cells. (**b**) Quantification of CD8 and CD4 expression by image J software. (**c**) The enlarged CLSM image of lung treated by dual MOF.

The effects of particles on in vivo T cell recruitment in lung metastasis were investigated in mice bearing B16F10 lung metastases. For tracking purposes, PB, MIL88, and dual MOF were labeled in advance with QD. Figure 6a displayed the CLSM images of lung metastases after the tumor-bearing mice were treated with PB, MIL88, and dual MOF at 24 h post injection. Several results can be drawn by the results. First, three systems can accumulate in lung, which might be attributed by margination and EPR (enhanced permeation and retention) effects of particles [12]. Second, all groups exhibited a strong expression of CD4^+^ and CD8^+^ (the typical markers of the upregulation of the T cell surface and indicated immune activity) (Figure 6b). The accumulation of T cells can be understood by the ROS generation in the lungs, in which Fe^2+^ ions of MOF reacted with H_2_O_2_ in tumor environment and inactive the organelles and proteins for the aberrant accumulation. Further activation of autophagy increases the antigens that could load into MHC class I and II to induce direct cytotoxic reaction and produce immunoprotective properties in the tumor. Figure 6c represented the enlarged images of CLSM exhibited CD4^+^ and CD8^+^ expression in lung metastasis tumor, indicating the good distribution of CD8^+^ expressed cells.

**Figure 7:**
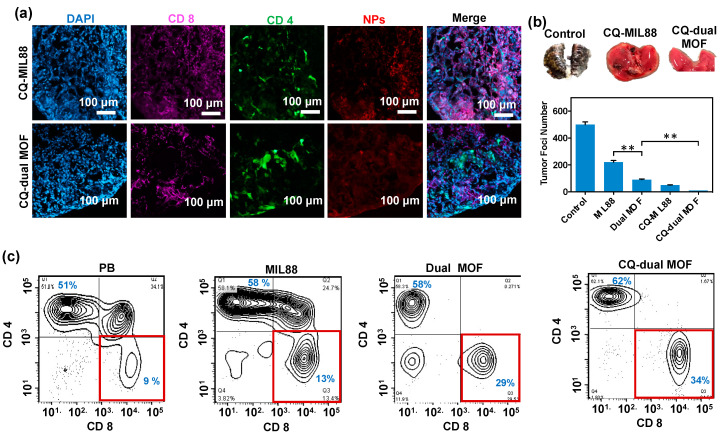
In vivo study of mice bearing B16F10 lung metastases treated with CQ-MIL88 and CQ- dual MOF. (**a**) CLSM images of lung metastases treated by CQ-MIL88 and CQ-dual MOF. (**b**) Images of dissected lung metastases treated by PBS (control), CQ-MIL88, and CQ-dual MOF. (**c**) Patterns of flow cytometry showing the CD4^+^ and CD8^+^ expression of T cells in lungs after various treatments. Quantitative significant statistical data were calculated via Student’s *t*-test, ** *p* < 0.01.

CQ-loaded MIL88 and dual MOF were injected intravenously to lung metastasis-bearing mice to estimate the eliciting of T cells. As shown in Figure 7a, CLSM images of lung metastases-bearing mice treated with CQ-MIL88 and CQ-dual MOF at 24 h post injection exhibited many strong CD4^+^ and CD8^+^ expressed cells. The significant improvement of T cells could be understood by the combination effects of CQ and Fenton reactions. As previous described, CQ trapped in lysosomal compartment would deplete hydrogen ions and change the internal environment. It blocked the degradation substrates in lysosomes and stopped the metabolization, inhibiting the self-protection of autophagosomes. Therefore, it caused cell death and released tumor-associated antigens to elicit T cells to tumors. With the recruiting of T cell, the metastatic suppression effects of CQ-MIL88 and CQ-dual MOF were investigated by injecting 100 μL of particles intravenously to lung metastasis-bearing mice at 7 days after tumor implantation. Subsequently, the tumor *foci* number was calculated after 14 days. The B16F10 metastasis is a type of aggressive tumors which prefers to colonize the distinct organs via circulation. The images in Figure 7b revealed the macroscopic appearance of lungs treated by CQ-MIL88 and CQ-dual MOF. Compared to control group, the numbers of tumor nodules were decreased. After calculation, there were ~440 tumor foci in the control group, but fewer than 10 tumor foci were observed after CQ-MIL88 and CQ-dual MOF, indicating the preliminary suppression of the tumor. Flow cytometry also displayed the relative population of T cells after various treatments (Figure 7c). Compared to the PB group, the CQ-dual MOF possessing the effects on programmed ROS generation and autophagy inhibition displayed a three-fold greater of CD8+ expression. Such effective T cell eliciting also reflected to tumor inhibition in Figure 7b. The gating strategy of T cells in flow cytometry was given in Figure S3. In the literature, CQ effectively blocked autophagy at the lysosomal degradation step in mouse breast cancer cell lines, and targeting autophagy-inhibited melanoma tumor growth by inducing a massive infiltration of immune cells, including NK cells and CD8+ into B16-F10 tumor cells [43,44]. Compared to their results, our systems integrated to dual MOF and dysfunctional autophagy could elicit higher numbers of T lymphocytes at the metastasis.

To understand the toxicity in vivo, both liver and kidney functions, including alanine aminotransferase (ALT), alkaline phosphatase (ALP), blood urea nitrogen (BUN), and serum creatinine (CRE), were evaluated 48 h post treatment. In brief, 100 μL of solution containing with 0.5 wt% MIL88 or dual MOF was injected into 6- to 8-week-old female C57BL/6 mice intravenously. Then, 48 h post treatment, an analysis of blood biochemistry (ALT, ALP, BUN, and CRE) was performed by 20 μL of whole blood withdrawn via submandibular through AmiShield (ProtectLife International Biomedical Inc., Taoyuan, Taiwan). Compared to the control group, only slight influence in liver and kidney functions was detected after treating MIL88 and dual MOF (Figure S4). On the other hand, despite the materials exhibiting potential in immune therapy, the limitations for translation into clinic need to be further overcome in the future. First, controlled drug release might be affected by tumor heterogeneity, including blood flow, pH variation, and oxygenation, leading to the decrease of responses and insufficient drug release. Second, the accumulation of materials in the tumor might also be limited because of the recognition of immune system and clearance of the reticuloendothelial or mononuclear phagocyte system. To translate the system to industrial application, the challenges in biological problems, scale-up of manufacturing, biosafety, regulations, and cost utility must be addressed.

## 4. Conclusions

A core-shell metal-organic framework nanocube composed of a Prussian blue core and Fe^2+^-containing metal-organic framework shell was developed to program reactive oxygen species and transported Chloroquine at a lung metastasis. The internalization-mediated cancer cell uptake exhibited the strong colocalization of nanocubes to cancer cells in vitro and in vivo. By co-delivering Chloroquine, the inhibition of autophagy through lysosomal deacidification was observed by MOF nanocubes, facilitating the block of the self-defense mechanism. At the tumor site, the core-shell nanocube with effective catalytic performance and dysfunctional autophagy elicited the ~3-fold infiltration of T lymphocytes. The synergy of T cell recruitment and ROS generation transported by dual MOF in the metastatic tumors inhibit the tumor foci in lung. Such cube-shaped nanocatalytics may provide a new avenue for lung disease therapy, potentially leading to the effective clinical immune therapy.

## Data Availability

Data are available upon reasonable request. All data relevant to the study are included in the article. The full data set is held by the corresponding author, please email with any requests for extra data.

## Supplementary Materials

The following supporting information can be downloaded at: https://www.mdpi.com/article/10.3390/pharmaceutics14030527/s1, Figure S1. Colloidal stability of PB, MIL88 and dual MOF in DMEM + 10% FBS over 24 h; Figure S2. CQ release patterns of PB, MIL88 and dual MOF; Figure S3. The gating strategy of T cells in flow cytometry; Figure S4. Liver function (ALT and ALP) and kidney function (BUN and CRE) at 48 h postinjection of PBS, MIL88 and dual MOF (n = 5).
