# Supplementary material for: Programmed Catalytic Therapy-Mediated ROS Generation and T-Cell Infiltration in Lung Metastasis by a Dual Metal-Organic Framework (MOF) Nanoagent
Source: Pharmaceutics. 2022 Feb 27;14(3):527. doi: 10.3390/pharmaceutics14030527 (PMC8955711; doi:10.3390/pharmaceutics14030527)
Supplement: Supplementary file 1 [file pharmaceutics-14-00527-s001.zip › pharmaceutics-1584846-supplementary.pdf]

pharmaceutics

MDPI

Supplementary Materials: Programmed Catalytic Therapy-Mediated ROS Generation and T-Cell Infiltration in Lung Metastasis by Dual Metal Organic Framework (MOF) Nanoagents

Bhanu Nirosha Yalamandala, Pin-Hua Chen, Thrinayan Moorthy, Thi My Hue Huynh, Wen-Hsuan Chiang and Shang-Hsiu Hu

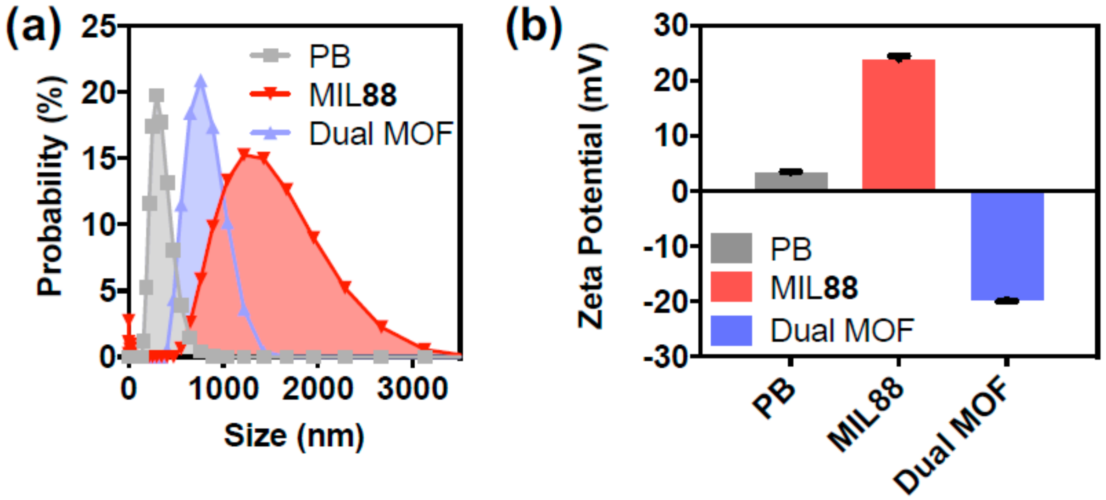

Figure S1. Colloidal stability of PB, MIL88 and dual MOF in DMEM + 10% FBS over 24 h.

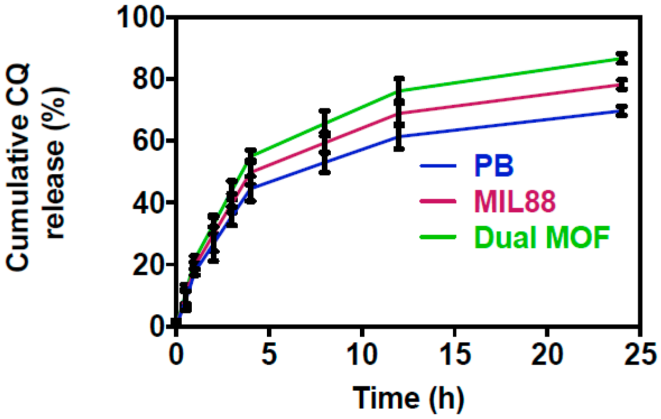

Figure S2. CQ release patterns of PB, MIL88 and dual MOF.

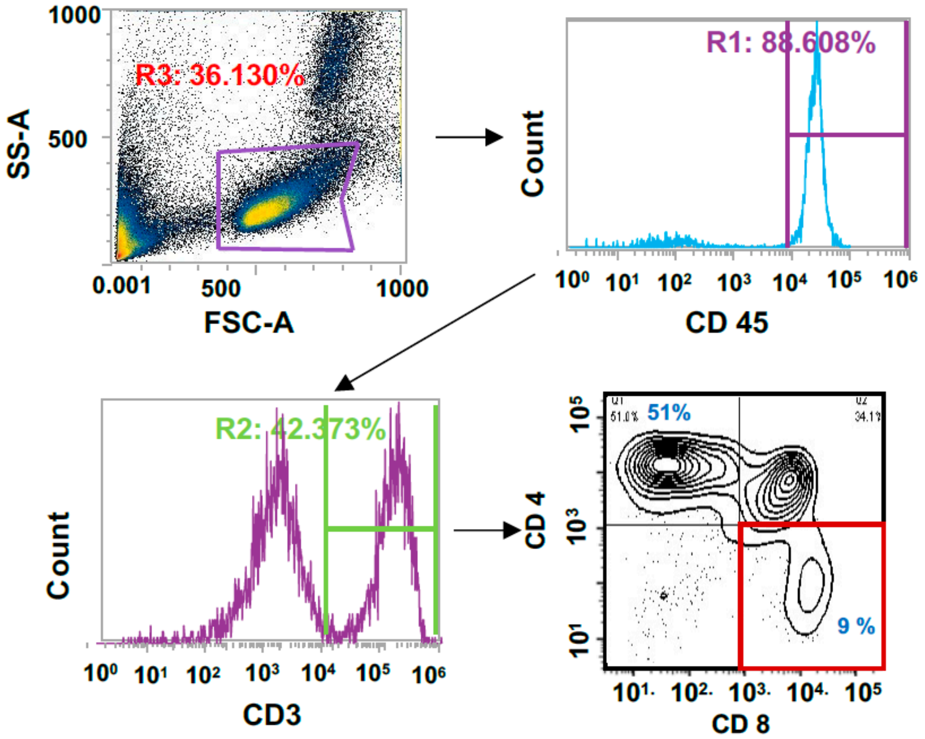

Figure S3. The gating strategy of T cells in flow cytometry.

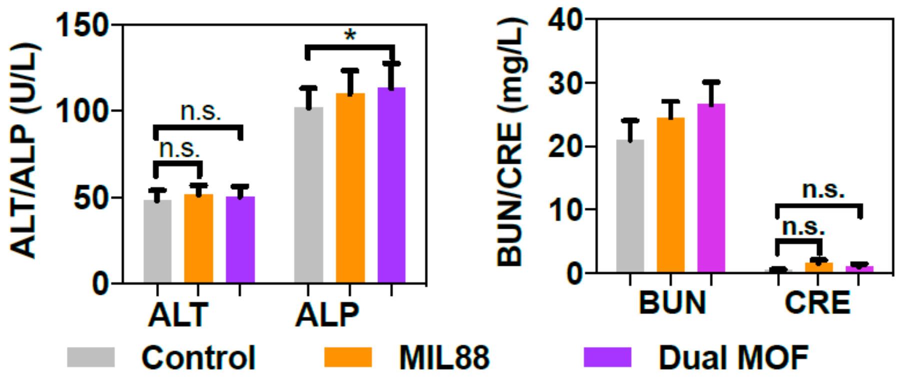

Figure S4. Liver function (ALT and ALP) and kidney function (BUN and CRE) at 48 h postinjection of PBS, MIL88 and dual MOF (n = 5).
